# Rubrofusarin Attenuates Chronic Restraint Stress-Induced Depressive Symptoms

**DOI:** 10.3390/ijms21103454

**Published:** 2020-05-13

**Authors:** Jee Hyun Yi, Jieun Jeon, Huiyoung Kwon, Eunbi Cho, Jeanho Yun, Young Choon Lee, Jong Hoon Ryu, Se Jin Park, Jong Hyun Cho, Dong Hyun Kim

**Affiliations:** 1 Center for Synaptic Brain Dysfunctions, Institute for Basic Science, Daejeon 169148, Korea; jeehyunyi@kaist.ac.kr; 2 Department of Medicinal Biotechnology, College of Health Sciences, Dong-A University, Busan 49315, Korea; ji6785@naver.com (J.J.); kwonhuiyoung@naver.com (H.K.); bee2634@naver.com (E.C.); yclee@dau.ac.kr (Y.C.L.); 3 Department of Biochemistry, College of Medicine, Dong-A University, Busan 49201, Korea; yunj@dau.ac.kr; 4 Department of Oriental Pharmaceutical Science, College of Pharmacy, Kyung Hee University, Seoul 02447, Korea; jhryu63@khu.ac.kr; 5 School of Natural Resources and Environmental Sciences, Kangwon National University, Chuncheon 24341, Korea; sejinpark@kangwon.ac.kr

**Keywords:** rubrofusarin, depressive disorder, chronic restraint stress, neuroinflammation, Akt, synaptic plasticity

## Abstract

The aim of this study was to examine whether rubrofusarin, an active ingredient of the Cassia species, has an antidepressive effect in chronic restraint stress (CRS) mouse model. Although acute treatment using rubrofusarin failed, chronic treatment using rubrofusarin ameliorated CRS-induced depressive symptoms. Rubrofusarin treatment significantly reduced the number of Fluoro-Jade B-positive cells and caspase-3 activation within the hippocampus of CRS-treated mice. Moreover, rubrofusarin treatment significantly increased the number of newborn neurons in the hippocampus of CRS-treated mice. CRS induced activation of glycogen synthase kinase-3β and regulated development and DNA damage responses, and reductions in the extracellular-signal-regulated kinase pathway activity were also reversed by rubrofusarin treatment. Microglial activation and inflammasome markers, including nod-like receptor family pyrin domain containing 3 and adaptor protein apoptosis-associated speck-like protein containing CARD, which were induced by CRS, were ameliorated by rubrofusarin. Synaptic plasticity dysfunction within the hippocampus was also rescued by rubrofusarin treatment. Within in vitro experiments, rubrofusarin blocked corticosterone-induced long-term potentiation impairments. These were blocked by LY294002, which is an Akt inhibitor. Finally, we found that the antidepressant effects of rubrofusarin were blocked by an intracerebroventricular injection of LY294002. These results suggest that rubrofusarin ameliorated CRS-induced depressive symptoms through PI3K/Akt signaling.

## 1. Introduction

Exposure to stress is known to affect various brain functions [1,2]. For instance, acute stress induces norepinephrine and glucocorticoid release from the adrenal glands, causing bodily changes, allowing individuals to adaptively respond to the stressor [3,4,5]. However, the experience of chronic stress is believed to be involved in the onset of depression [6,7]. Constant feelings of low self-esteem and anhedonia are the main symptoms of depression. Different forms of stress can induce different depression conditions [8,9]. Although the exact mechanisms underlying the onset of depression are unknown, many drugs for patients with depression were developed based on the theory that levels of monoamine neurotransmitters are low in these patient’s brains [10,11]. However, current antidepression drugs not only are not fully effective for patients but also possess severe side effects [12,13,14]. Thus, developing antidepressants is of importance.

Monoamine neurotransmitters, including serotonin, dopamine, and norepinephrine, are involved in various psychological aspects such as mood, emotion, arousal, and compensation. Insufficient levels of monoamine neurotransmitters are related to the pathophysiology underlying depression [15,16]. Most frequently prescribed antidepressants currently include Prozac (fluoxetine) and Zoloft (sertraline). These are selective serotonin reuptake inhibitors which were developed based on the monoamine theory, where other food and drug administration (FDA)-approved antidepressants are also based on the monoamine theory. However, these antidepressants have various side effects such as nausea, loss of sexual desire, and insomnia [17]. Moreover, studies revealed that antidepressants reduce depressive symptoms in 50% of patients and prevent relapses in 23% of patients [18]. Therefore, new antidepressants should be developed for better treatment efficacy.

Rubrofusarin, which is isolated from the Cassia species, is a member of the class of benzochromenones. Rubrofusarin has been reported to have anticancer, antibacterial, and antioxidant effects [19,20,21]. Recently, we found that rubrofusarin prevents amyloid-β (Aβ) aggregation and improves Alzheimer’s disease-like dementia [22]. Moreover, recent studies reported that rubrofusarin inhibits monoamine oxidase-A, which can degrade norepinephrine, serotonin, and dopamine, with good blood–brain barrier penetration value [23]. These suggest that rubrofusarin may increase monoamine neurotransmitter levels in the brain and demonstrate antidepressant-like effects. Thus, this study aimed to test the effects of rubrofusarin on the onset of depressive symptoms in a chronic restraint stress-induced depression-like model and mode of action.

## 2. Results

### 2.1. Chronic Rubrofusarin Administration Ameliorated Chronic Restraint Stress (CRS)-Induced Depressive Symptoms

**Figure 1:**
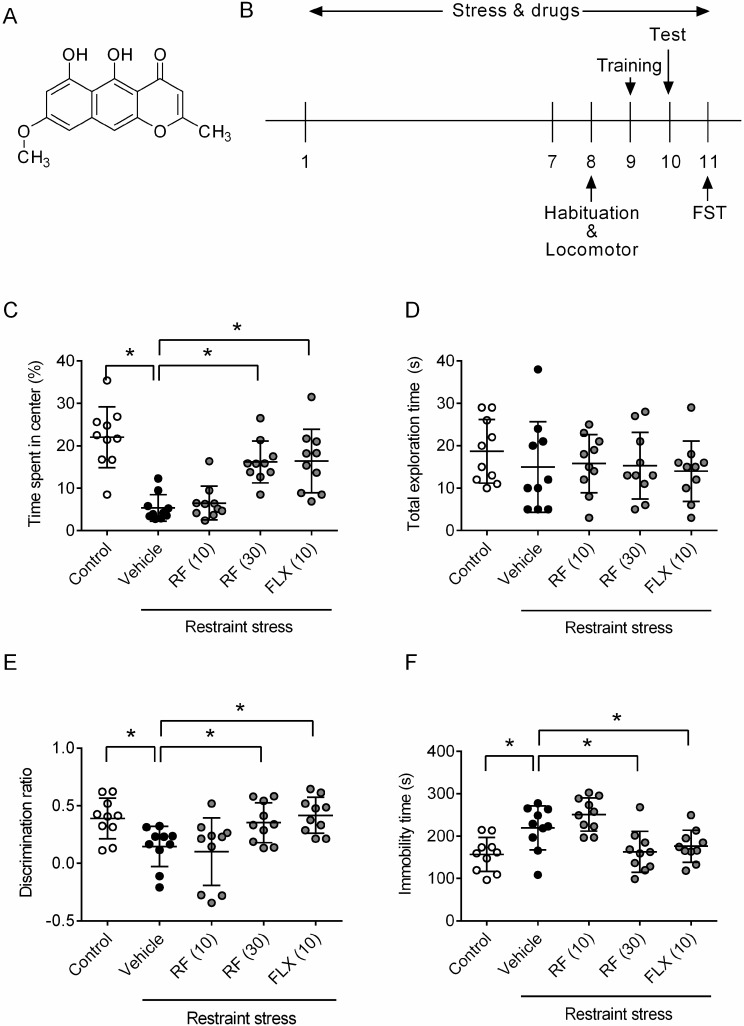
Effect of rubrofusarin on depressive symptoms: Restraint stress (4 h per day) was treated for 11 days. Rubrofusarin (10 or 30 mg/kg) or fluoxetine (10 mg/kg) was administered to mice 1 h before the restraint stress treatment. (**A**) Structure of rubrofusarin. (**B**) Schematic diagram of experimental schedule. (**C**) Time spent in the center of an open field in the habituation trial of object recognition test. (**D**) Total exploration time for objects in training trial of object recognition test. (**E**) Discrimination ration in the test trial of an object recognition test. (**F**) Immobility time in forced swimming test. Data represented as mean ± SD with raw data. * *p* < 0.05.

To examine the effect of rubrofusarin (RF, Figure 1A) on CRS-induced depressive symptoms, we administered rubrofusarin to mice 1 h before the restraint stress for 11 days. Mice experienced restraint stress for 4 h a day for 11 days (Figure 1B). To test the effect of rubrofusarin on CRS-induced cognitive dysfunction, an object recognition test was conducted. During a habituation session, the CRS-treated group showed significantly shorter exploration time in the center zone of the open field compared to control group. This suggested that CRS increased anxiety. RF (30 mg/kg) or fluoxetine (FLX, 10 mg/kg)-treated CRS groups showed no significant changes in the time spent in the center zone compared to the control group (F4,45 = 5.619, *p* < 0.05, *n* = 10/group, Figure 1C), suggesting reductions in anxiety. During the training session, there were no significant differences in the total exploration time for the two objects between groups (F4,45 = 0.475, *p* > 0.05, *n* = 10/group, Figure 1D). During the test session, the CRS group showed a significantly lower discrimination index compared to the control group, suggesting potential impairment in recognition memory. RF (30 mg/kg) or FLX (10 mg/kg)-treated CRS groups showed significantly higher discrimination ratios compared to the CRS group (F4,45 = 5.648, *p* < 0.05, *n* = 10/group, Figure 1E). During the forced swimming test for testing levels of depression, the CRS group showed significantly higher immobility time compared to the control group. RF (30 mg/kg) or FLX (10 mg/kg)-treated CRS groups showed significant reductions in immobility time compared to the CRS group (F4,45 = 3.733, *p* < 0.05, *n* = 10/group, Figure 1F). These behavioral results suggest that RF reduced CRS-induced depressive symptoms including anxiety, memory impairments, and overall levels of depression.

### 2.2. Chronic Rubrofusarin Administration Rescued CRS-Induced Hippocampal Neuronal Damage

**Figure 2:**
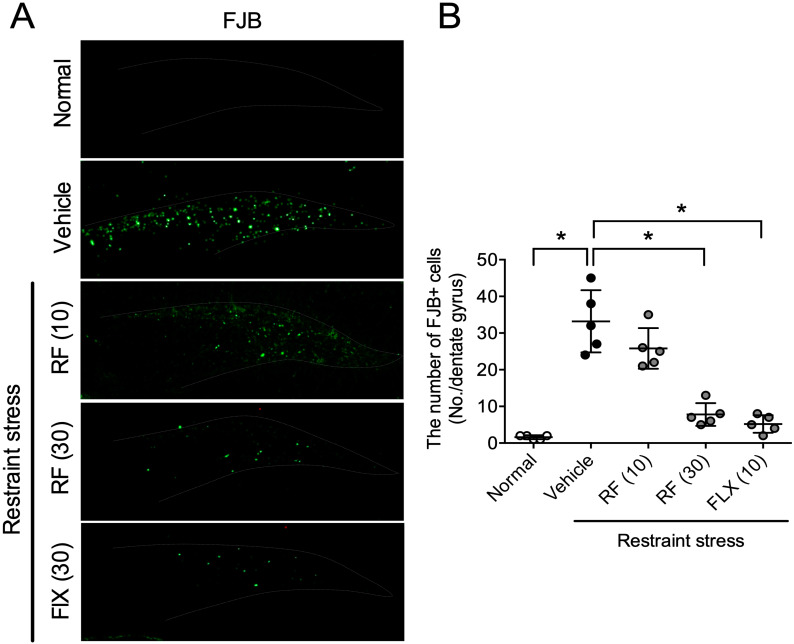
Effect of rubrofusarin on neurodegeneration: (**A**) Photomicroscopic image of Fluoro Jade-B (FJB)-positive cells in the dentate gyrus region. (**B**) Quantitative analysis of the number of FJB-positive cells in the dentate gyrus region. Data are represented as mean ± SD with raw data. * *p* < 0.05.

Previous reports indicated that CRS causes neurodegeneration in the hippocampus [24]. To test whether RF rescues CRS-induced neuronal cell death, we conducted Fluoro Jade-B (FJB) staining (Figure 2A). Mice were sacrificed 1 h after the forced swimming test (FST) test for FJB staining. The CRS-treated group showed significantly more FJB-positive cells in the hippocampal dentate gyrus (DG) region (*p* < 0.05). RF (30 mg/kg) or FLX (10 mg/kg) treatment blocked the increase of FJB-positive cells by CRS (F4,20 = 41.01, *p* < 0.05, *n* = 5/group, Figure 2B). The FJB results suggest that RF blocked CRS-induced neuronal cell death in the hippocampus.

### 2.3. Chronic Rubrofusarin Administration Blocked CRS-Induced Reduction of Adult Neurogenesis

**Figure 3:**
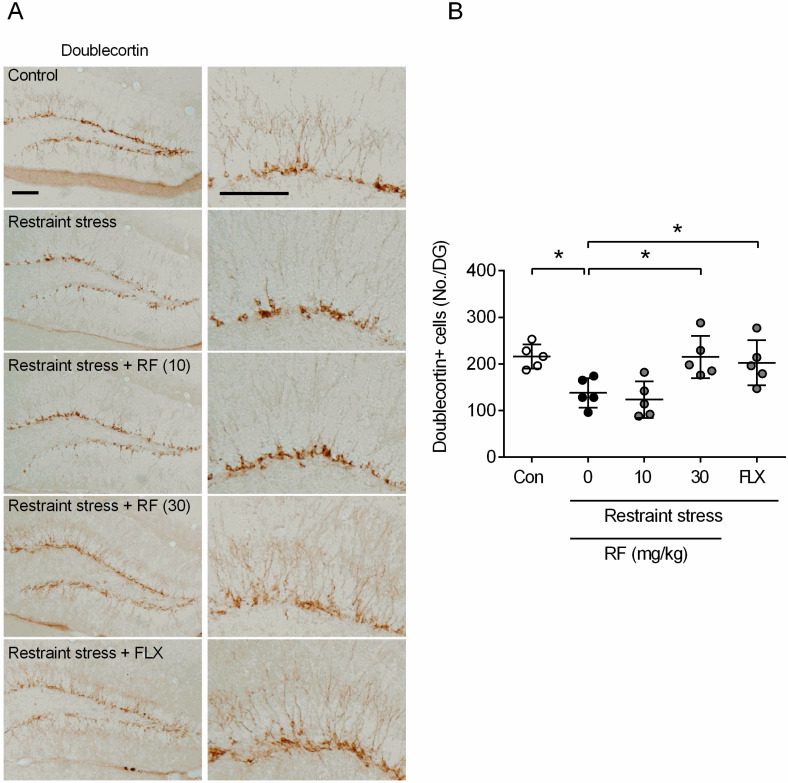
Effect of rubrofusarin on adult neurogenesis: (**A**) Photomicroscopic image of doublecortin-positive cells in the dentate gyrus region. Bar = 100 µm. (**B**) Quantitative analysis of the number of doublecortin-positive cells in the dentate gyrus region. Data represented as mean ± SD with raw data. * *p* < 0.05.

Various animal models and patients with major depressive disorders show reductions in adult neurogenesis within the hippocampus [25]. To test whether rubrofusarin ameliorates reductions in neurogenesis using the CRS model, we measured doublecortin-positive cells in the hippocampus. Mice were sacrificed 1 h after the FST test for doublecortin immunostaining. The CRS-treated group showed significant reductions in the number of doublecortin-positive cells in the DG region of the hippocampus (*p* < 0.05, Figure 3A,B). RF treatment ameliorated this reduction in a dose-dependent manner (F4,20 = 9.615, *p* < 0.05, *n* = 5/group, Figure 3B). FLX also ameliorated the reduction in the number of doublecortin-positive cells in CRS-treated mice (*p* < 0.05, Figure 3A,B).

### 2.4. Chronic Rubrofusarin Administration Blocked CRS-Induced Neuroinflammation

**Figure 4:**
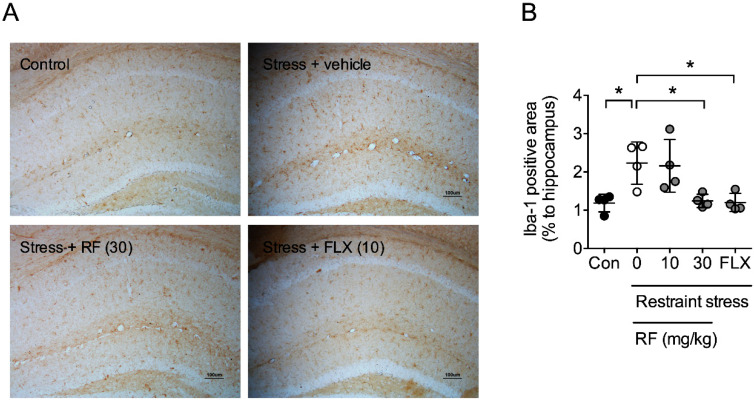
Effect of rubrofusarin on neuroinflammation: (**A**) Photomicroscopic image of Iba-1-immunopositive cells in the hippocampus. Bar = 100 µm. (**B**) Quantitative analysis of Iba-1-immunopositive area in the hippocampus. Data represented as mean ± SD with raw data. * *p* < 0.05.

Inflammation within the CNS is a component of depression [26,27]. Therefore, we observed the effect of RF on CRS-induced neuroinflammation. It was found that CRS induced microglial activation in the hippocampus (Figure 4A,B). RF (30 mg/kg) and FLX (10 mg/kg) significantly suppressed CRS-induced microglial activation (F4,15 = 6.367, *p* < 0.05, *n* = 4/group, Figure 4B). The immunohistochemical result suggests that RF blocked CRS-induced neuroinflammation.

### 2.5. Chronic Rubrofusarin Administration Blocked CRS-Induced Synaptic Dysfunctions

**Figure 5:**
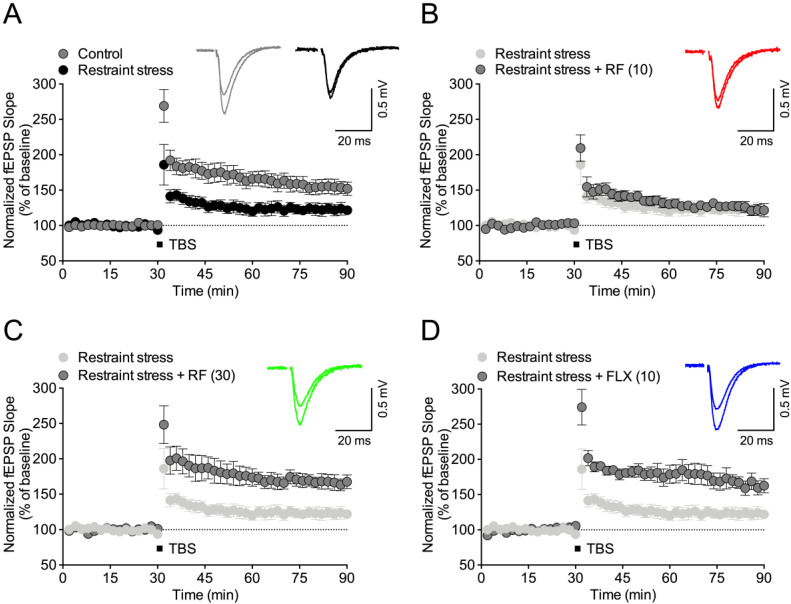
Effect of rubrofusarin on synaptic deficit: Long-term potentiation in the Schaffer-collateral pathway was induced by 2 trains of theta burst stimulation (TBS, 5 trains of 4 pulses at 100 Hz) after 30 min stable baseline. (**A**) Normalized Field excitatory postsynaptic potential (fEPSP) slop of control and chronic restraint stress (CRS) group for 90 min. (**B**) Normalized fEPSP slop of CRS + rubrofusarin (10 mg/kg) group for 90 min. (**C**) Normalized fEPSP slop of control and CRS + rubrofusarin (30 mg/kg) group for 90 min. (**D**) Normalized fEPSP slop of CRS + fluoxetine (10 mg/kg) group for 90 min. Data represented as mean ± SD.

Synaptic dysfunctions in the brain of patients with depression were reported [28]. Therefore, we tested the effect of RF on synaptic dysfunction in the CRS-treated depression model. Long-term potentiation (LTP) was significantly reduced in the hippocampus of the CRS-treated group (*p* < 0.05, Figure 5A). This reduction was ameliorated by RF (Figure 5B,C) or FLX (Figure 5D) treatment (*p* < 0.05). These results suggest that RF blocked CRS-induced synaptic deficits.

### 2.6. Rubrofusarin Blocked Corticosterone-Induced LTP Impairment Through Regulating the Akt Signaling

**Figure 6:**
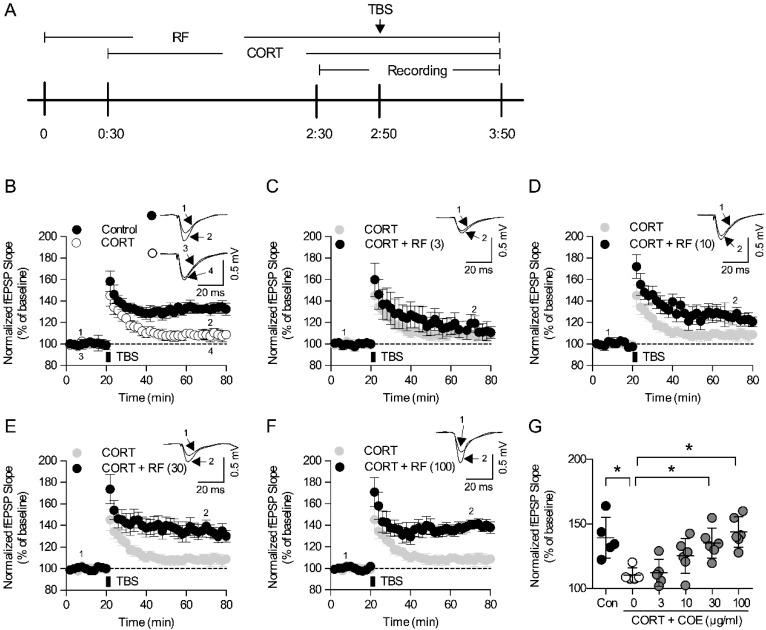
Effect of rubrofusarin on corticosterone-induced synaptic deficit: (**A**) Schematic diagram of experimental schedule. Hippocampal slices were treated with rubrofusarin (RF) from 30 min before corticosterone (CORT) treatment. HFS was delivered to the slices 30 min after the CORT treatment. (**B**) Normalized fEPSP slop of control and CORT (1 µM) group for 80 min. (**C**) Normalized fEPSP slop of CORT + RF (3 µM) group for 80 min. (**D**) Normalized fEPSP slop of CORT + RF (10 µM) group for 80 min. (**E**) Normalized fEPSP slop of CORT + RF (30 µM) group for 80 min. (**F**) Normalized fEPSP slop of CORT + RF (100 µM) group for 80 min. Data represented as mean ± SD. (**G**) Normalized fEPSP slop of each group during last 10 min. Data represented as mean ± SD with raw data. * *p* < 0.05.

**Figure 7:**
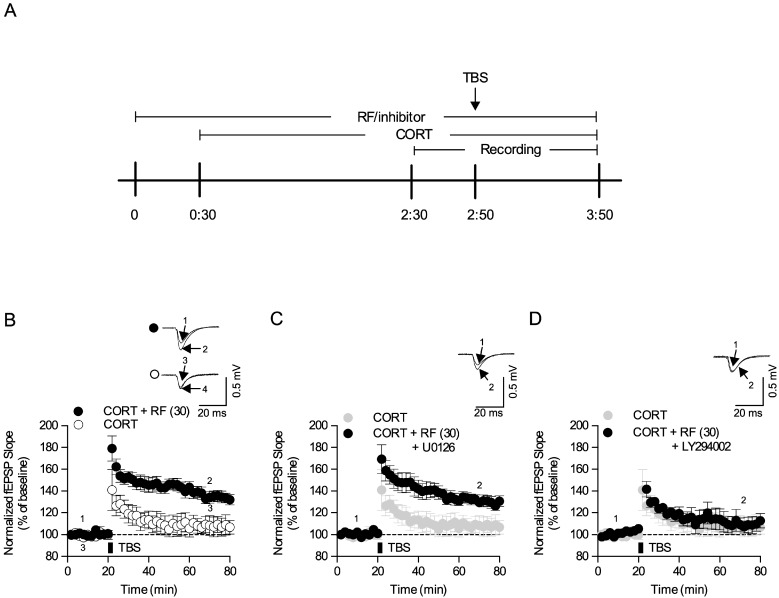
The role of Akt/GSK-3β signaling in the effect of rubrofusarin synaptic deficit: (**A**) Hippocampal slices were co-treated with rubrofusarin (RF) and inhibitors. Corticosterone (1 µM, CORT) was introduced to the slices 30 min after RF treatment. Theta burst stimulation (TBS, 5 trains of 4 pulses at 100 Hz) was delivered to the slices 30 min after the CORT treatment. (**B**) Normalized fEPSP slop of CORT + RF (30 µM) and CORT group for 80 min. (**C**) Normalized fEPSP slop of CORT + RF (30 µM) + U0126 (50 µM) group for 80 min. (**D**) Normalized fEPSP slop of CORT + RF (30 µM) + LY294002 (50 µM) group for 80 min. Data represented as mean ± SD.

To examine the underlying mechanism of rubrofusarin, we tested rubrofusarin in corticosterone-induced LTP impairments in the acute hippocampal slices (Figure 6A). Corticosterone (CORT, 1 µM) suppressed Theta burst stimulation (TBS)-induced LTP induction (Figure 6B,G). RF (30 and 100 µM) blocked this CORT-induced LTP deficit in a concentration-dependent manner (F5,27 = 7.453, *p* < 0.05, *n* = 5–6/group, Figure 6C–G). Moreover, suppression of Akt signaling with LY294002 but not ERK signaling with U0126 blocked the effect of RF (*p* < 0.05, Figure 7A–D). These results suggest that Akt signaling might be required for the effect of RF on CORT-induced LTP impairment.

### 2.7. Akt Signaling Was Required for the Antidepressive Effect of Rubrofusarin

**Figure 8:**
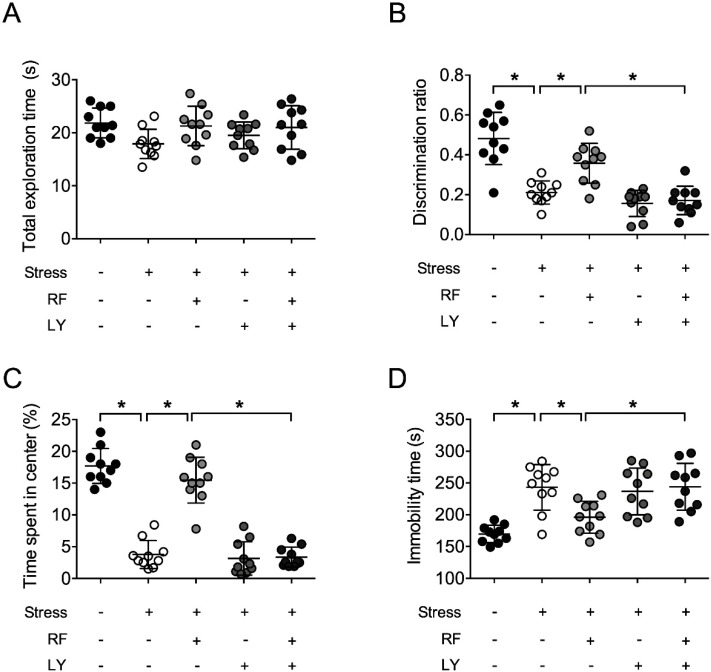
Effect of LY294002 on the effect of rubrofusarin on depressive symptoms: Mice were treated with LY204002 (1 nmol, 3 µL, intracerebroventricular injection (i.c.v.)) 30 min before rubrofusarin treatment. Rubrofusarin was administered to mice 1 h before restraint stress protocol. (**A**) Total exploration time for objects in training trial of object recognition test. (**B**) Discrimination ration in test trial of object recognition test. (**C**) Time spent in center of open field in the habituation trial of object recognition test. (**D**) Immobility time in forced swimming test. Data represented as mean ± SD with raw data. * *p* < 0.05.

To test whether Akt/glycogen synthase kinase-3 (GSK-3) signaling is involved in the antidepressive effects of rubrofusarin, LY294002 (LY) was injected into the third ventricle through the cannulae to block Akt/GSK-3-signaling. Neither treatments affected total exploration time during training session of the object recognition test (F4,45 = 2.419, *p* > 0.05, *n* = 10/group, Figure 8A). The effect of RF on stress-induced decreases of the discrimination ratio was blocked with LY (F4,45 = 24.77, *p* < 0.05, *n* = 10/group, Figure 8B). The effect of RF on stress-induced reductions in time spent in the center zone of the open field was also blocked by LY (F4,45 = 75.53, *p* < 0.05, *n* = 10/group, Figure 8C). In terms of immobility time during the forced swimming test, LY blocked the effect of RF, as well (F4,45 = 11.60, *p* < 0.05, *n* = 10/group, Figure 8D). These behavioral results suggest that the antidepressive effect of RF might require activation of Akt/GSK-3 signaling.

**Figure 9:**
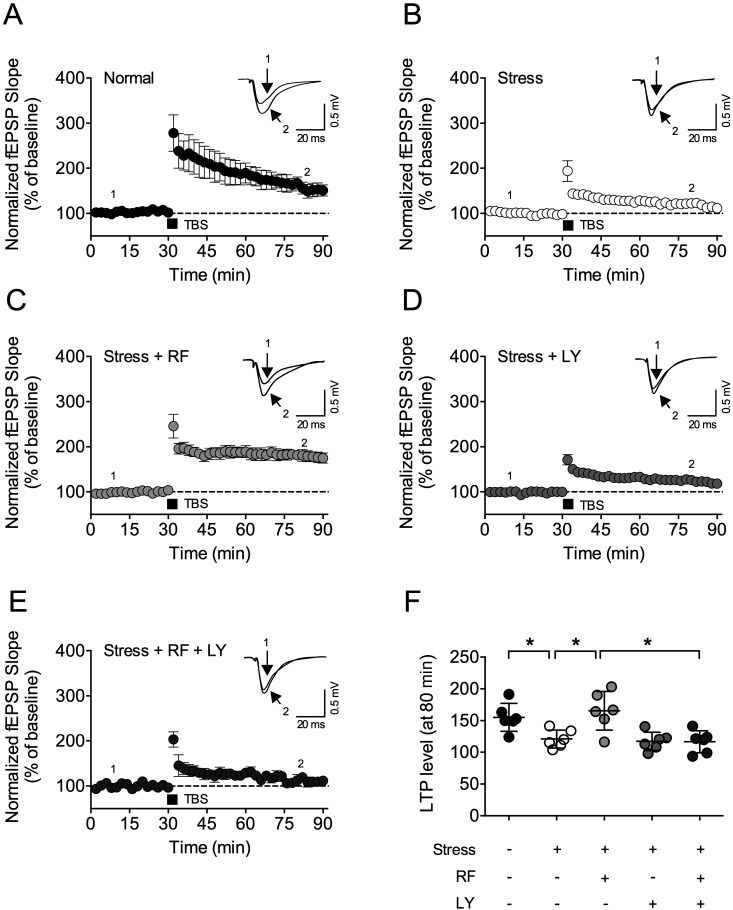
Effect of LY294002 on the effect of rubrofusarin on synaptic deficit: Long-term potentiation in the Schaffer-collateral pathway was induced by theta burst stimulation (TBS, 5 trains of 4 pulses at 100 Hz) after 30 min stable baseline. (**A**) Normalized fEPSP slop of normal group for 90 min. (**B**) Normalized fEPSP slop of CRS (stress) group for 90 min. (**C**) Normalized fEPSP slop of stress + RF (30 mg/kg) group for 90 min. (**D**) Normalized fEPSP slop of stress + LY294002 (LY, 1 nmol, 3 µL, i.c.v.) group for 90 min. (**E**) Normalized fEPSP slop of stress + RF (30 mg/kg) + LY (1 nmol, 3 µL, i.c.v.) group for 90 min. Data represented as mean ± SD. (**F**) Normalized fEPSP slop of each group during last 10 min. Data represented as mean ± SD with raw data. * *p* < 0.05.

To test whether Akt/GSK-3-signaling is also required for the effects of rubrofusarin on stress-induced synaptic deficits, LTP in the hippocampus was measured (Figure 9A–F). LY treatment blocked RF-restoring LTP impairment in the stress-treated hippocampus (F4,25 = 7.738, *p* < 0.05, *n* = 7 (7 slices from 3–4 mice)/group, Figure 9A–F).

## 3. Discussion

In the present study, we found that chronic treatment of rubrofusarin ameliorated CRS-induced depressive symptoms. Rubrofusarin blocked CRS-induced neurodegeneration and reduction in neurogenesis within the hippocampus. CRS-induced neuroinflammation was blocked by rubrofusarin. Dysfunctions in hippocampal synaptic plasticity were also rescued by rubrofusarin treatment. Within the in vitro experiments, rubrofusarin blocked corticosterone (CORT)-induced LTP impairments through regulating Akt signaling. Additionally, Akt inhibition blocked rubrofusarin-ameliorated CRS-induced depressive symptoms.

Stress is thought to be a major environmental trigger for individuals with clinical depression [29,30]. Stress can stimulate release of norepinephrine and glucocorticoid from the adrenal glands, which induce stress-related coping responses [31,32]. However prolonged stress, which can induce constant high levels of blood glucocorticoid and long-term stimulation in nervous system by norepinephrine, can cause harmful effects in the brain [33,34]. Pharmacological long-term elevations in corticosterone can induce depressive symptoms including depressive behaviors and neurochemical changes in mice. Glucocorticoid receptor (GR) antagonist blocked stress-induced depressive symptoms [35,36]. However, direct regulation of steroid receptors may cause unpredictable side effects including abnormal negative feedback of steroid hormones.

Chronic stress induced neuronal degeneration [37,38]. Although the precise mechanism is not clear, prolonged increases of corticosterone levels may induce abnormal activation of the *N*-methyl-d-aspartate receptor (NMDAR), which can increase intracellular Ca^2+^ levels [39,40]. Increased intracellular Ca^2+^ levels may activate caspase-3 through calpain, a Ca^2+^-sensing protease [41,42], thereby triggering neuronal death signals. In the present study, we found that CRS induced neuronal degeneration in the hippocampal DG region. Moreover, CRS increased active-caspase-3 levels in the hippocampus. These results suggest that CRS may induce neuronal degeneration and that this might be related to caspase-3 activation in the hippocampus. Rubrofusarin blocked CRS-induced neuronal degeneration.

Neurogenesis is believed to be the mechanism underlying learning and memory in the hippocampus [43,44]. Neurogenesis is decreased in various animal models of depression and within the depressed patient’s brain [45,46]. Chronic stress can suppress neurogenesis through suppression of phosphoinositide 3-kinases (PI3K)/Akt signaling [47]. Activation of GSK-3β is also involved in stress-related suppression of neurogenesis [48,49]. Lithium, a GSK-3 inhibitor, showed antidepressive effects and restored neurogenesis [50]. Moreover, many FDA-approved antidepressants including fluoxetine and venlafaxine also restored suppressed neurogenesis in depression models, suggesting that regulation of neurogenesis is an important action of antidepressants [51]. In the present study, we found that CRS suppressed neurogenesis and that rubrofusarin blocked this in the hippocampus.

Neuroinflammation is additionally a major underlying mechanism of depression. Chronic stress-activated microglia have been studied as key mediators for the onset of brain diseases [52,53]. Recent studies show that the nod-like receptor family pyrin domain containing 3 (NLRP3) inflammasome is upregulated in microglia and may play an important role in depression [54]. Moreover, studies show NLRP3 inflammasome activation in animal models of depression [55] as well as in patients with depression [56]. Silymarin showed antidepressive effects through regulation of NLRP3 inflammasome activation [57,58]. P2X7 receptor and NLRP inflammasome activation in the hippocampus were involved in chronic stress-induced onset of depressive symptoms [58]. Glucocorticoid-induced NLRP3 inflammasome activation in the hippocampal microglia might mediate chronic stress-induced depressive symptoms [55]. These suggest that the NLRP3 inflammasome might be a novel target for antidepressants. In the present study, CRS induced microglial activation and increased NLRP3 inflammasomes in the hippocampus. Rubrofusarin suppressed CRS-induced these neuroinflammation.

Recently, it was found that corticosterone suppressed hippocampal synaptic plasticity through activation of caspase-3 [59]. Chronic glucocorticoid receptor (GR) activation may induce synaptic depression through caspase-3 activation, which might be mediated by abnormal activation of NMDAR [60,61]. GR-induced synaptic depression may be a phenomenon of the depressed brain [6,62,63]. Antidepressants ameliorated this synaptic depression through various mechanisms. In the present study, CRS caused synaptic deficits in the hippocampus. Oral administration of rubrofusarin blocked CRS-induced synaptic deficits. Moreover, corticosterone, by itself, caused synaptic deficits within the hippocampal slices. Rubrofusarin ameliorated corticosterone-induced synaptic deficits through regulation of Akt/GSK-3β signaling.

Rubrofusarin blocked various CRS-induced depressive symptoms including behavioral and synaptic deficits and neuroinflammation. These results may indicate that rubrofusarin has antidepressive, anti-neurodegenerative, and anti-neuroinflammation effects. Otherwise, rubrofusarin may act as a blocker of upper stream signals, which are activated by CRS, such as GR [64,65]. In the present study, we found that Akt/GSK-3β signaling is implicated in the various effects of rubrofusarin. GR activation can suppress Akt, resulting in GSK-3β activation [59,66]. Activation of GSK-3β is implicated in neurogenesis [67], synaptic deficits [68], and neuroinflammation [69]. Therefore, rubrofusarin inhibited GSK-3β resulting in amelioration of various symptoms including neurogenesis, synaptic deficits, and neuroinflammation.

In conclusion, rubrofusarin blocked CRS-induced onset of depressive disorder through inhibiting Akt-relating signaling. These results suggest that rubrofusarin may be a needed compound for developing antidepressant. However, we still do not know whether rubrofusarin inhibits Akt activity directly or indirectly. To elucidate this, further study will be needed.

## 4. Materials and Methods

### 4.1. Animals

CD-1 mice weighing 25–30 g (male, 6-week-old) were purchased from Samtako (Osan, Korea). The mice were habituated to the living environment for 1 week before each experiment. Five mice were housed in one cage and were provided water and food ad libitum (temperature, 23 ± 1 °C; humidity, 60 ± 10%) under a 12 h illumination cycle (lights on from 07:30 to 19:30). Animal treatments and maintenance were carried out in accordance with the Animal Care and Use Guidelines issued by Dong-A University, Republic of Korea. All experimental protocols using mice were approved by the Institutional Animal Care and Use Committee of Dong-A University (approval number, DIACUC-approve-19-33, 10/12/2019).

### 4.2. Restraint Stress

Using clear plastic tube (3 cm in diameter and 10 cm in length) having many holes for ventilation, restraint stress was introduced to mice for 4 h per day for 11 days restraint from 10:00 a.m. to 2:00 p.m. Mice are able to move anterior limbs and the head but not the body. The control group (nonrestraint mice) stayed in the home cages until the object recognition test started. Behavioral tests were started 1 h after the 4-h period of restraint stress.

### 4.3. Objective Recognition Memory Test

Habituation of mice to open field (cube shape with 25 cm^3^) was conducted for 10 min. During habituation, time spent in center (5 cm × 5 cm) was measured for analyzing anxiety state of the mice. Thirty minutes later, mice were placed in the same open field with two same objects (two glass boxes). Total exploration time was measured during training. After 24 h, mice were return to the same open field for testing. The two different objects (glass box and crystal cylinder) were again present. Mice were allowed to freely explore the environment and the objects for 5 min. Total time spent exploring the novel (Tnovel) and familiar (Tfamiliar) objects were measured. Discrimination ratio was calculated by the following formula: Tnovel − Tfamiliar/(Tnovel + Tfamiliar).

### 4.4. Forced Swimming Test

Using a clear glass cylinder (25 cm in height and 14 cm in diameter) containing water (24 ± 2 °C), the forced swimming test was performed for 6 min in a dim environment. Their immobility times were recorded using the video-based Ethovision System (Noldus Information Technology B.V., Wageningen, Netherlands) during the last 4 min of the 6 min test.

### 4.5. Tissue Slices Preparation

Mice were anesthetized using isoflurane (3%) immediately after the forced swimming test. Transcardial perfusion was conducted with 100 mM phosphate buffered saline (PBS, pH 7.4) followed by ice-cold 4% paraformaldehyde. Then, isolated brains were postfixed in PBS containing 4% paraformaldehyde for 12 h. Then, the brains were stored in a sucrose solution (30% in 50 mM PBS, 4 °C). Coronal sections including the hippocampus were stored in a storage solution at 4 °C.

### 4.6. Floro-Jade B Staining

Fluoro-Jade B staining was conducted to label degenerating cells. Coronal sections mounted on gelatin-coated slide glass were incubated in 0.06% potassium permanganate for 15 min. The sections were rinsed for 1 min in distilled water (DW) and then incubated in the Fluoro-Jade B staining solution (0.0001% wt/vol in distilled water containing 0.1% acetic acid) for 30 min and rinsed with DW for 3 times. The slides were incubated in 100% of xylene and then coverslipped with Dibutylphthalate Polystyrene Xylene mounting media (Sigma-Aldrich, Saint Louis, MO, USA). The number of Fluoro-Jade–B-positive cells in the hippocampal dentate gyrus was measured. Only positive neurons with a near-complete cell body shape and size were counted. Cell counts were expressed as the total number of Fluoro-Jade-positive cells per section.

### 4.7. Immunohistochemistry

Primary antibodies including goat anti-doublecortin or rat anti-iba-1 antibody (1:1000 dilution) were mixed with 0.3% Triton X-100 and 1.5% normal serum. Free-floating sections were incubated for 24 h in PBS (4 °C) containing primary solution. After washing, the sections were treated with biotinylated secondary antibody (1:1000 dilution) for 90 min and then incubated in an avidin–biotin–peroxidase complex (1:100 dilution, 1h, room temperature). After washing, the sections were incubated in 3, 3′-diaminobenzidine solution (0.02% in DW containing 0.01% H_2_O_2_, 3 min). Finally, the sections were mounted on gelatin-coated slides. Then the slides were dehydrated in an ascending alcohol series (75%, 90%, 95%, and 100%) and cleared in xylene. Only near-complete cell body shape and size were counted. Cell counts were expressed as the total number of doublecortin-positive cells per section. Iba-1-immunopositive area was analyzed with ImageJ software.

### 4.8. Hippocampal Slices Preparation and Electrophysiology

Artificial cerebrospinal fluid (ACSF) is composed with NaCl (124 mM), KCl (3 mM), NaHCO_3_ (26 mM), NaH_2_PO_4_ (1.25 mM), CaCl_2_ (2 mM), MgSO_4_ (1 mM), and d-glucose (10 mM). We rapidly isolated the hippocampus and submerged it in chilled ACSF. For tissue slicing, we used McIlwain tissue chopper; 400-µm-thick hippocampal slices were incubated in ACSF (20–25 °C, 2 h) before the experiment.

Field excitatory postsynaptic potential (fEPSP) was recorded in the CA1 area (Schaffer-collateral-commissural pathway). Constant stimuli were delivered through stimulating electrode (0.033 Hz). The slope of the evoked fEPSP was averaged over consecutive recordings evoked at 30 s intervals; 30 min after the initiation of a stable baseline, theta burst stimulations (TBS: 5 trains of 4 pulses at 100 Hz) were introduced to induce long-term potentiation (LTP). LTP was quantified by comparing the mean fEPSP slope at 80 min after the TBS with the mean fEPSP slope during the baseline period.

### 4.9. Microinfusion of Drugs

Mice were placed in a stereotaxic frame (David Kopf Instruments, Los Angeles, CA, USA) under isoflurane anesthesia (induction 3% and maintenance 2%), and guide cannulae (26 G) was aimed at the right lateral ventricle (AP, −0.02 mm from bregma; ML, −0.80 mm from midline; DV, −2.50 mm from the dura) based on atlas of the mouse brain [70]. The guide cannulae were fixed to the skull with dental cement and covered with dummy cannulae. Following surgery, mice were allowed to recover for 7 days. LY294002 was dissolved in 0.1% dimethyl sulfoxide in saline (0.9% NaCl) before infusion. Fifteen minutes before the oral administration of rubrofusarin, mice were carefully restrained by hand and infused with LY294002 (1 nmol, 3 µL) or vehicle through injector cannulae (30 G) extended 1.0 mm beyond the tips of guide cannulae. The infusion volume was 3 µL, and the infusion rate was 0.5 µL/min. After the infusion, the infusion needle was left in the guide cannula for 1 min to ensure proper delivery of the reagents.

### 4.10. Statistics

The results of all experiments were analyzed with one-way ANOVA followed by Turkey’s test for multiple comparisons. Student’s t-test was used to compare two groups. The values are expressed as the means ± SD with raw data; *p* < 0.05 was considered significant.

## Abbreviations

CRS	Chronic restraint stress
REDD1	Regulated in development and DNA damage responses 1
NLRP3	Nod-like receptor family pyrin domain containing 3
GSK-3	Glycogen synthase kinase-3
PI3K	Phosphoinositide 3-kinases
ACSF	Artificial cerebrospinal fluid
fEPSP	Field excitatory postsynaptic potential
TBS	Theta burst stimulation
LTP	Long-term potentiation
RF	Rubrofusarin
FLX	Fluoxetine
DG	Dentate gyrus
CORT	Corticosterone
NMDAR	*N*-methyl-d-aspartate receptor
GR	Glucocorticoid receptor
